# Myeloperoxidase and Advanced Oxidation Protein Products in the Cerebrospinal Fluid in Women and Men with Parkinson’s Disease

**DOI:** 10.3390/antiox11061088

**Published:** 2022-05-30

**Authors:** Emilio Fernández-Espejo, Fernando Rodríguez de Fonseca, Ana Luisa Gavito, Antonio Córdoba-Fernández, José Chacón, Ángel Martín de Pablos

**Affiliations:** 1 Reial Acadèmia de Medicina de Catalunya, 08001 Barcelona, Spain; 2 Laboratorio de Medicina Regenerativa, Hospital Regional Universitario, 29010 Málaga, Spain; ana.gavito@ibima.eu; 3 Unidad de Gestión Clínica de Salud Mental, Instituto de Investigación Biomédica de Málaga (IBIMA), Hospital Regional Universitario, 29010 Málaga, Spain; 4 Departamento de Podología, Universidad de Sevilla, 41009 Sevilla, Spain; acordoba@us.es; 5 Servicio de Neurología, Hospital Quirónsalud Infanta Luisa, 41010 Sevilla, Spain; jchaconp2@gmail.com; 6 Departamento de Cirugía, Universidad de Sevilla, 41009 Sevilla, Spain; amartin8@us.es; 7 Unidad de Anestesiología y Reanimación, Servicio de Cirugía, Hospital Macarena, 41009 Sevilla, Spain

**Keywords:** myeloperoxidase, advanced oxidation protein products, cerebrospinal fluid, Parkinson’s disease, duration

## Abstract

Background: Myeloperoxidase (MPO) and advanced oxidation protein products, or AOPP (a type of MPO-derived chlorinated adducts), have been implicated in Parkinson´s disease (PD). Human MPO also show sex-based differences in PD. The objective was to study the relationship of MPO and AOPP in the cerebrospinal fluid (CSF) with motor features of idiopathic PD in male and female patients. Methods: MPO concentration and activity and AOPP content were measured in the CSF and serum in 34 patients and 30 controls. CSF leukocytes and the integrity of the blood-brain barrier were evaluated. Correlations of MPO and AOPP with clinical variables were examined. Results: The blood-brain barrier was intact and CSF leukocyte count was normal in all patients. CSF MPO concentration and activity were similar in the cohort of patients and controls, but CSF MPO content was significantly higher in male patients than in PD women (*p* = 0.0084). CSF MPO concentration correlated with disease duration in male and female patients (*p* < 0.01). CSF MPO concentration was significantly higher in men with disease duration ≥12 years versus the remainder of the male subjects (*p* < 0.01). Changes in CSF MPO in women were not significant. Serum MPO concentration and activity were significantly higher in all PD patients relative to controls (*p* < 0.0001). CSF MPO was not correlated with serum MPO. Serum AOPP were detected in all patients, but CSF AOPP was undetectable in 53% of patients. AOPP were not quantifiable in controls. Conclusions: CSF MPO is not a good biomarker for PD because mean CSF MPO concentration and activity are not different between the cohort of patients and controls. CSF MPO concentration positively correlated with disease duration in men and women, but CSF MPO is significantly enhanced only in male patients with disease duration longer than 12 years. It can be hypothesized that the MPO-related immune response in early-stage PD might be weak in all patients, but then the MPO-related immune response is progressively enhanced in men, not women. Since the blood-brain barrier is intact, and CSF MPO is not correlated with serum MPO, CSF myeloperoxidase would reflect MPO content in brain cells, not blood-derived cells. Finally, serum AOPP was detected in all patients, but not controls, which is consistent with the occurrence of chlorinative stress in blood serum in PD. The study of CSF AOPP as biomarker could not be assessed because the ELISA assay was hampered by its detection limit in the CSF.

## 1. Introduction

The enzyme myeloperoxidase (MPO) belongs to the family of heme-peroxidases, which are essential components of the immune defense system. This defensive function is based on the generation of microbicidal compounds through two biochemical pathways: the halogenation and peroxidation cycles. Antimicrobial molecules are powerful oxidants and pro-inflammatory molecules [1,2,3,4,5], and over-activity of heme-peroxidases has been implicated in the oxidative and inflammatory damage of neurons and tissues, which subserves neurodegeneration [6,7,8,9,10,11,12,13,14,15].

MPO is a normal constituent in granules of peripheral neutrophils and monocytes/macrophages as well as in brain glial cells (astrocytes and microglia). MPO biochemical relies on the production of hypochlorite, hypochlorite-modified proteins, chlorinated amines, and other reactive species [2,4,5,16,17,18]. MPO, which is expressed by neurons in the *substantia nigra pars compacta* and contributes to α-synuclein pathology, has been implicated in Parkinson’s disease (PD) [10,12,19,20,21,22]. Increased MPO expression in surviving neurons and activated glial cells in the *substantia nigra* in diseased patients and animal models of PD has been reported [10,22,23,24,25]. The elimination of MPO ameliorates the motor and lesional nigrostriatal damage and reduces the presence of hypochlorite-modified proteins within the *substantia nigra*, in the MPTP animal model of PD [10,26]. In addition, sex-based differences in the response of MPO-positive microglia and astrocytes are reported in the experimental models of PD [27,28,29], and gender differences in MPO activity exist in healthy subjects [30,31]. Since the risk of developing Parkinson´s disease is twice as high in men than women [29], it would be of great interest discerning if there are sex differences regarding MPO content and activity in PD patients.

Among hypochlorite-modified proteins, Advanced Oxidation Protein Products or AOPP are suggested to play a role in neuroinflammation and oxidation processes in PD, and they are closely linked to MPO [13,32,33,34,35,36]. AOPP content is correlated with serum MPO in pathological conditions such as diabetes or uremia [32,33], and it is worth studying if these hypochlorite-modified adducts are linked to changes in MPO in PD.

Brain MPO and hypochlorite-modified derivatives have been proposed to be the target for antiparkinsonian treatment [10,13,22,34,37,38,39] but, to date, no studies have examined how myeloperoxidase and AOPP in the cerebrospinal fluid (CSF) are related with the motor features of idiopathic PD. The objective of this study was to look at the relationship of MPO and AOPP in the CSF with demographic and clinical features of the disease, as well as to discern if gender differences subserve changes in MPO. The CSF is in close contact with brain tissue, and it reflects the biochemical changes associated with neurodegeneration [40,41].

## 2. Materials and Methods

### 2.1. Participants

This prospective, observational, and cross-sectional study was carried out at Hospital Macarena, Sevilla, Spain. Thirty-four patients and 30 controls were evaluated after careful selection. Patients had age-at-PD onset of 45–75 years, and they were diagnosed of PD if they presented rigidity, bradykinesia, and resting tremor (classic motor signs of Parkinsonism), as well as a reliable loss of dopamine-transporter signal on dorsal striatum [41,42,43,44,45]. Dopaminergic loss was measured with ^123^I-Ioflupane DAT-SPECT by expert physicians from the Service of Nuclear Medicine [43,44,45]. Patients with atypical deficits or family members with PD were ruled out to exclude hereditary forms of Parkinsonism. Control participants were recruited from volunteers subjected to intradural anesthesia before surgery. Control participants were group-matched by age and sex to PD subjects. They were excluded if they had a first-degree relative with PD or another neurological disorder. CSF was collected before anesthesia, and surgery and anesthesia themselves had no impact on the parameters to be analyzed.

### 2.2. Clinical Information

Demographic information was obtained from patients and controls: Age, gender, body mass index (BMI), and years of education. Clinical data included the modified Hoehn–Yahr staging, the International Parkinson and Movement Disorder Society-Sponsored revision of the Unified Parkinson’s Disease Rating Scale (MDS-UPDRS), the modified Schwab–England activities of daily living scale, age-at-PD onset, disease duration in years, and the antiparkinsonian medication regimen. Antiparkinsonian medication (which includes levodopa, dopamine agonists, and supportive medication that enhances dopaminergic effects) was expressed as levodopa equivalent daily dose (LEDD, mg), according to established formula [45,46,47,48].

Individuals suffering from renal, hematological, liver, and cardiovascular dysfunctions, as well as malabsorption, morbid obesity, dementia, autoimmune diseases, acquired immunodeficiency syndrome, cancer, infectious conditions or taking anti-inflammatory drugs or statins were excluded from the study. MPO and AOPP levels in the CSF can be altered in these conditions [49,50]. Morbid obesity was diagnosed when BMI was higher than 35 kg/m^2^. All participants were non-smokers, non-alcohol drinkers, and non-coffee drinkers, according to established criteria [50,51,52,53].

### 2.3. Biofluid Collection, ELISA Analysis and Albumin Index

MPO and AOPP were measured in the CSF. In addition, to further discern whether CSF MPO and AOPP are related to blood molecules, serum MPO and AOPP were also evaluated. CSF was collected by physicians by using lumbar puncture. Two ml of CSF were collected, aliquoted, coded, and rapidly frozen at −80 °C. A 0.5-mL fresh collection was employed to observe the absence of traumatic puncture, and to quantify red cells (CSF with >500 red cells/microL was discarded). Five ml of blood was collected by cephalic vein puncture to obtain serum with a serum separator tube (BD Vacuotainer). Serum was centrifuged at 2500 rpm during 10 min, and then it was immediately aliquoted, and frozen at −80 °C in 0.5 mL aliquots. CSF and serum aliquots were unfrozen and sonicated with homogenizing solution (50 mM HEPES, 150 mM NaCl, 0.6µm leupeptin, 1 mM phenylmethylsulfonil fluoride, 1% Triton X-100, pH 7.4). Myeloperoxidase and AOPP concentration were evaluated with commercially available Enzyme-linked Immunosorbent Assay kits (Human Myeloperoxidase ELISA Kit, ref. ab119605, Abcam, Cambridge, UK; Oxyselect^TM^ AOPP assay kit, ref. STA-318, Cells Biolab Inc., San Diego, CA, USA), following the manufacturer’s instructions. The sensitivity of the ELISA assay with human biofluids is ~10 pg/mL (MPO) and ~0.1 µM (AOPP). Myeloperoxidase activity was measured with an Enzyme activity kit (Myeloperoxidase Activity Assay Kit, Colorimetric, ab105136, Abcam), following manufacturer’s instructions. This assay kit can be used to detect myeloperoxidase as low as 0.010 mU per mL. The reaction period was 10 min. Each sample was analyzed in duplicate (undiluted CSF; 1/50 dilution, serum).

The albumin index was calculated to discard that CSF levels of MPO or AOPP are related to the alteration of the blood-brain barrier [40,45]. For this purpose, one ml of blood was collected by cephalic vein puncture, in gel-coated serum separator tubes (BD Vacuotainer, Barcelona, Spain). The samples were centrifuged at 1500 rpm during 20 min to separate clot and trapped cells, and the serum was aliquoted, coded, and frozen at −80 °C in 0.5 mL aliquots. Albumin was quantified by standard immunochemical nephelometry in CSF and serum samples (SIEMENS BN II system), and the albumin index was calculated as follows = CSF albumin content (mg/dL)/serum albumin content (g/dL). An albumin index below 9 is considered as normal. The number of leukocytes per µL was counted in CSF samples by using a Neubauer´s chamber to discern that there is no infection or infiltration of blood leukocytes in the cerebrospinal fluid [49].

### 2.4. Statistics and Ethics

Quantitative variables were compared with the Student’s *t*-test, and comparisons of dichotomous variables were carried out with the χ^2^ test. Correlations between variables were carried out with the Pearson’s test. Correlation value was adjusted for age, age-at-PD onset, gender, BMI, and education. Data normalization was verified with the Shapiro–Wilk test. The patients were classified into subgroups according to the Hoehn–Yahr staging (early-stage disease, stages 1, 1.5, and 2; moderate-advanced disease, stages 2.5, 3, and 4; no patients at stage (5), and these subgroups were compared. Ninety-five percent confidence interval and sample size power were calculated to avoid making Type II error, and the statistical power was set at β = 0.2 or >80% (α = 0.01).

Protocols were approved by the internal ethics and scientific boards of Junta de Andalucia (PEIBA, CEI Sevilla #20151-0520554-1 and #2017-121418738), Hospital Universitario Macarena (CEI #19/05/2010 and CEI #2149-29/10/2013), and University of Seville (CEE-27/05/2010 and Seccion Investigacion/Gd-21/06/2010). The subjects consent was obtained according to the Declaration of Helsinki (BMJ 1991; 302:1194).

## 3. Results

### 3.1. Demographic and Clinical Parameters

Demographic and CSF analytical data were not found to be different between controls and patients with PD, as shown in Table 1. The albumin index was lower than 9 in every subject, indicating that there is not breakdown of the blood-brain barrier in PD patients. Mean leukocyte count was similar in all groups. The clinical parameters of PD patients are shown in Table 1.

### 3.2. Myeloperoxidase and Advanced Oxidation Protein Products in the CSF and Serum

#### 3.2.1. Cohort of Patients and Controls’

**Figure 1:**
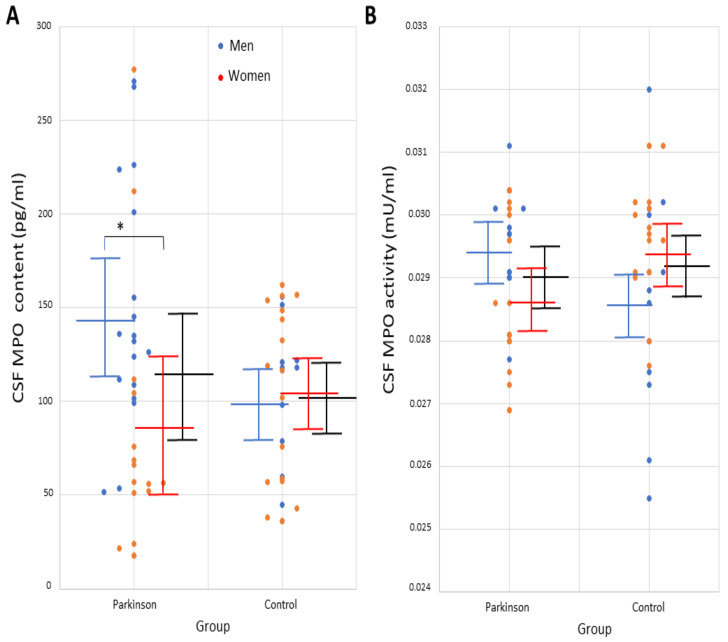
Individual values of (**A**) myeloperoxidase concentration (pg/mL) and (**B**) MPO activity (mU/mL) in the cerebrospinal fluid in male and female patients with idiopathic Parkinson’s disease and controls. Mean and standard deviation are represented with solid lines (black lines, cohort of patients and control participants; men are represented in blue and women in orange). * *p* < 0.01. Abbrev.: CSF, cerebrospinal fluid; MPO, myeloperoxidase.

Regarding the entire group of patients and controls, CSF MPO concentration and activity was not significantly different between the patients and controls, as shown in Table 1 and Figure 1. On the other hand, serum MPO concentration and activity were significantly higher in the patients relative to controls (concentration, t = 4.3568; activity, t = 5.5387, *p* < 0.0001) (Table 1). Based on MPO levels, 95% confidence interval and post-hoc sample size power was calculated. In the CSF study, 95% confidence interval was of 89.7 to 138.9 (concentration) and 0.0286 to 0.0294 (activity). In the serum study, post-hoc sample size power was 100%. Regarding AOPP, these adducts were detected in 16 out of 34 CSF samples in the patients (47%), and they were undetectable in control subjects. The detection limit of the ELISA assay was a major limitation for quantifying AOPP content in patients’ CSF. On the other hand, all serum samples presented quantifiable levels of AOPP in the patients, which were otherwise undetectable in the control serum (Table 1).

CSF MPO concentration and activity significantly correlated with each other (r = +0.471, *p* = 0.0049, Table 2). A significant correlation was found between CSF MPO concentration, not activity, and disease duration (r = +0.475, *p* = 0.0045, Table 2). To further analyze effects on disease duration, the cohort of patients was divided into subjects with disease duration ≥12 years, and participants with PD duration under 12 years (mean duration of the PD cohort was 12.1 years). CSF MPO concentration was observed to be significantly higher in the cohort of patients with disease duration ≥12 years (149 ± 85 pg/mL, *n* = 14) than in the remainder patients (86 ± 44 pg/mL, *n* = 20; t = 2.8288, *p* = 0.008) and controls (men, 101 ± 43 pg/mL, *n* = 30, t = 2.5021, *p* = 0.01).

No significant correlations between CSF MPO concentration or activity and other parameters such as serum MPO content or disease severity (as measured with rating scales) were observed (Table 2). Thus, CSF MPO concentration was found not to be significantly different in patients at early-stage PD (114 ± 73 pg/mL, *n* = 23) relative to patients with moderate-advanced PD (108 ± 64 pg/mL, *n* = 11). CSF MPO activity was found not to be significantly different in patients with early PD (0.0289 ± 0.001 mU/mL, *n* = 23) relative to patients with moderate-advanced PD (0.0291 ± 0.001 mU/mL, *n* = 11). Finally, AOPP correlations were not studied because AOPP content in CSF samples was below the detection limit of the ELISA assay in many patients, as explained.

#### 3.2.2. Study of Sex-Based Differences

CSF MPO concentration was significantly higher in male patients than in women with PD (t = 2.9161, *p* = 0.0064), although no differences with controls were found, as shown in Table 3. Demographic and clinical parameters of all men and women are shown in Table 3. Serum MPO content and activity in men was significantly higher in PD patients than in controls (MPO content, t = 5.0074, *p* = 0.0054; MPO activity, t = 5.6278, *p* < 0.0001). Besides, serum MPO content and activity in women was significantly higher in PD patients than in controls (MPO content, t = 4.6580, *p* < 0.0001; MPO activity, t = 4.8684, *p* < 0.0001; Table 3). No other differences were found. Individual values of MPO concentration and activity in the CSF in men and women are shown in Figure 1. Serum AOPP was detected in every patient but, in the CSF, the ELISA assay was hampered by its detection limit, as explained (Table 3).

**Figure 2:**
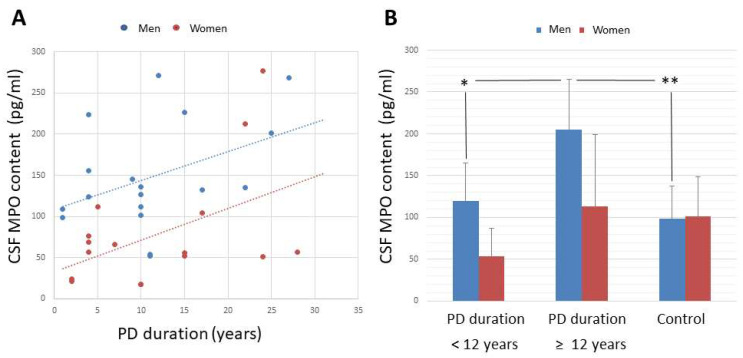
(**A**) Correlation between MPO level in the CSF and duration of Parkinson’s disease, and (**B**) CSF MPO concentration (pg/mL) in male and female patients with disease duration shorter than 12 years, and patients with PD duration ≥ 12 years. (**A**) CSF MPO concentration correlated with disease duration in both male and female patients (men, r = +0.469, *p* = 0.0496, *n* = 18; women, r = +0.565, *p* = 0.0226, *n* = 16). (**B**) CSF MPO concentration was observed to be significantly higher in male patients with disease duration ≥12 years (206 ± 61 pg/mL, *n* = 11) than in the remainder male patients (119 ± 45 pg/mL, *n* = 7), and control men (98 ± 39 pg/mL, *n* = 13). Mean ± standard deviation, * *p* < 0.01, ** *p* < 0.001. Abbrev.: CSF, cerebrospinal fluid; MPO, myeloperoxidase; PD, Parkinson’s disease.

After analyzing correlations, CSF MPO concentration correlated with disease duration in both subgroups (men, r = +0.469, *p* = 0.0496, *n* = 18; women, r = +0.565, *p* = 0.0226, *n* = 16; Figure 2A, Table 4). CSF MPO concentration and activity significantly correlated with each other in both male and female patients (men, r = +0.470, *p* = 0.0490; women, r = +0.498, *p* = 0.0496, Table 4).

As for effects of disease duration, CSF MPO concentration was observed to be significantly higher in male patients with disease duration ≥12 years (206 ± 61 pg/mL, *n* = 11) than in the remainder male patients (119 ± 45 pg/mL, *n* = 7; t = 3.2397, *p* = 0.0051), and control men (98 ± 39 pg/mL, *n* = 13, t = 5.2505, *p* < 0.0001, Figure 2). Significant differences were not found in female patients because, although CSF MPO content is progressively enhanced in women (disease duration <12 years, 51 ± 32 pg/mL; disease duration ≥12 years, 115 ± 91 pg/mL), no significant differences among subgroups and female controls were found (103 ± 47 pg/mL, *n* = 17).

**Figure 3:**
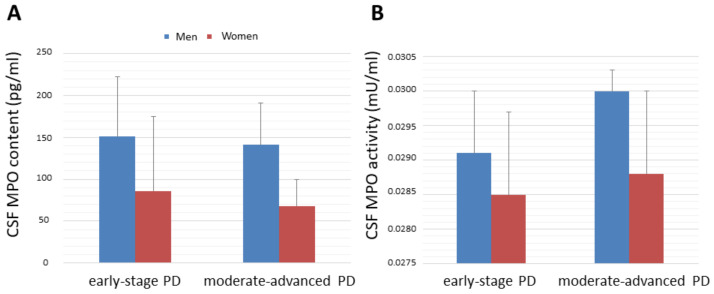
(**A**) MPO concentration (pg/mL) and (**B**) MPO activity (mU/mL) in the CSF in male patients and female ones at early-stage disease (Hoehn and Yahr stages 1 and 2) and patients with moderate-advanced disease (Hoehn and Yahr stages 2.5–4). No significant differences were found. Mean ± standard deviation. Abbrev.: CSF, cerebrospinal fluid; MPO, myeloperoxidase; PD, Parkinson´s disease.

No significant correlations between CSF MPO concentration or activity and other parameters such as disease severity or serum MPO content were observed (Table 4). CSF MPO concentration was found not to be significantly different in male and female patients at early-stage PD (men, 151 ± 71 pg/mL, *n* = 12; women, 87 ± 89 pg/mL, *n* = 10) relative to patients with moderate-advanced PD (men, 141 ± 49 pg/mL, *n* = 6; women, 68 ± 21 pg/mL, *n* = 6). CSF MPO activity was found not to be significantly different in male and female patients at early-stage PD (men, 0.0291 ± 0.001 mU/mL, *n* = 12; women, 0.0284 ± 0.001 pg/mL, *n* = 10) relative to patients with moderate-advanced PD (men, 0.0301 ± 0.0003 mU/mL, *n* = 6; women, 0.0289 ± 0.001 mU/mL pg/mL, *n* = 6). The lack of influence of disease severity on CSF MPO concentration or activity in men and women is illustrated in Figure 3.

## 4. Discussion

The enzyme myeloperoxidase, which is expressed by microglia and neurons in the *substantia nigra pars compacta* and contributes to α-synuclein pathology [21,22,54], holds promise as a biomarker in PD [19,20]. However, according to our findings, CSF MPO would not be a good biomarker for prognostication or diagnosis of PD, because mean MPO content and activity are similar in the cohort of PD patients and healthy controls. In addition, CSF MPO concentration or activity are not correlated with disease severity or serum MPO. The lack of correlation between MPO in the CSF and serum MPO suggests that both molecules are independent to each other and do not interact on pathophysiological levels in PD [41].

A novel finding of this study is that CSF MPO concentration is correlated with disease duration in male and female patients. MPO concentration is significantly augmented in the CSF in men with disease duration longer than 12 years. However, in female patients, there is a progressive increase in CSF MPO content but differences with the control women are not significant. Therefore, MPO is reliably upregulated in the CSF in male patients with PD, not women, and after long duration of the disease. It can be hypothesized that the MPO-related immune response at early-stage PD might be weak in all patients with PD, but the MPO-related immune response and recruitment of MPO-positive cells would be progressively enhanced in men, not in female patients. In women, the MPO-related immune response seems to be weak throughout the disease.

Regarding sex-based differences, it is known that female patients have a higher mortality rate and faster progression of PD [55], and women report more problems than men in most clinical dimensions of the disease [56]. Our data indicate that the CSF MPO content in women is lower than that of men and, in contrast to male patients, it is not reliably increased throughout the disease. In this context, sex-based differences on the immune response of MPO-positive microglia and astrocytes are reported in experimental models of PD [27,28,29], and Hanamsagar, et al. have demonstrated that male microglia are more mature than female microglia [57]. The findings of this study could be related to differences in the immune response in women versus men. Further studies are required to support these hypotheses.

Many authors have reported that MPO is over-expressed in postmortem brain tissue [22,25,39], and the findings of this study support that MPO would be over-expressed in PD brain in male patients, not women, at advanced stages of the disease. The cellular localization of MPO in PD brain is a matter of discussion. On the one hand, several authors have observed increased MPO expression in surviving neurons and activated glial cells in the *substantia nigra* in PD [22,25]. On the other hand, Gellhaar, et al. [39] report that it is the number of MPO-positive blood-derived cells rather than glial cells that are increased in nigrostriatal regions of PD brains. Since the blood-brain barrier is found to be intact in PD patients, the leukocyte count in the CSF is normal, and CSF MPO does not correlate with serum MPO, the influence of blood MPO or infiltration of blood immune cells across the blood-brain barrier can be ruled out. Therefore, CSF MPO would reflect the enzyme content of MPO-positive brain cells rather than blood-derived immune cells.

In contrast to CSF MPO, serum MPO is significantly enhanced in all patients, confirming previous findings [58]. The findings suggest that the response of peripheral MPO-positive immune cells seems to be stronger than that of central immune cells in PD. Since the main biochemical function of MPO is to catalyze the formation of hypochlorite and chlorinated species, powerful pro-oxidative and inflammatory compounds [5,7,19,21,54,58,59,60], our results would highlight the importance of MPO in the oxidation and inflammation processes that subserve PD pathogenesis [23,24,25,58].

Another novel finding is that advanced oxidation protein products are detected in blood serum in all PD patients but not in controls. In the CSF, the ELISA assay was hampered by its detection limit, thus challenging its reliability. AOPP are pro-inflammatory chlorinated products that are created through the reaction of hypochlorite and chloramines with proteins [32,33,61,62,63]. AOPP are found in pathological conditions that exhibit phagocyte activation and chlorinative stress, a type of oxidative stress that is characterized by excess amounts of hypochlorite and derivatives [32,33,61,62,63]. Chlorinative stress and neuroinflammation have been implicated in the pathogenesis of PD [9,10,15,19,21,34,54], and the findings indicate the occurrence of chlorinative stress in blood in human PD.

The study has some limitations that should be acknowledged. We cannot infer causation, because it is a cross-sectional design, not a longitudinal study. The cohorts of participants were relatively small, and the detection threshold of the ELISA kit was a major limitation for quantifying AOPP concentration in the CSF. The results must be confirmed by means of larger samples in future studies, and the use of more sensitive methods. The strengths of our study include well-characterized patients with PD, rigorous data collection and selection of participants, the analysis of biofluids that are in close contact with brain tissue, and the measurement of concentration and activity of myeloperoxidase.

## 5. Conclusions

CSF MPO is not a good biomarker for PD because mean CSF MPO concentration and activity are not different between the cohort of patients and controls. CSF MPO concentration positively correlated with disease duration in men and women, but CSF MPO is significantly enhanced only in male patients with a disease duration longer than 12 years. It can be hypothesized that the MPO-related immune response in early-stage PD might be weak in all patients, but then the MPO-related immune response is progressively enhanced in men, not women. Since the blood-brain barrier is intact, and CSF MPO is not correlated with serum MPO, CSF myeloperoxidase would reflect MPO content in brain cells, not blood-derived cells. Finally, serum AOPP was detected in all patients, but not controls, which is consistent with the occurrence of chlorinative stress in blood serum in PD. The study of CSF AOPP as a biomarker could not be assessed because the ELISA assay was hampered by its detection limit in the CSF.

## Figures and Tables

**Table 1:** Demographic and clinical parameters in the cerebrospinal fluid and serum in the cohort of patients with PD and control participants.

Parameters	PD (*n* = 34)	Control (*n* = 30)	*p*
Age, years	65 ± 12	57.4 ± 14	NS
Gender, male *n*(%)	18 (60)	13(45)	NS
Body mass index, kg/m^2^	23.2 ± 3	24.6 ± 3	NS
Education, years	17.3 ± 1.6	17.4 ± 3.5	NS
Albumin index	6.9 ± 1.9	6.6 ± 1.6	NS
Leukocyte count, cells/µL	3.1 ± 0.7	2.7 ± 0.6	NS
CSF MPO content, pg/mL	114 ± 73	101 ± 43	NS
CSF MPO activity, mU/mL	0.0290 ± 0.001	0.0292 ± 0.001	NS
Serum MPO content, pg/mL	3770 ± 2871	1430 ± 683	<0.0001
Serum MPO activity, mU/mL	44.1 ± 6	35.1 ± 7	<0.0001
CSF AOPP content, µM	23.4 ± 29 (*n* = 16)	nd	
Serum AOPP content, µM	535 ± 349	nd	
Levodopa equivalent daily dose, mg	686.2 ± 532		
Disease duration, years	12.1 ± 7.8		
Age at PD onset, years	51.2 ± 14		
Modified Hoehn–Yahr stage	2.1 ± 0.7		
Modified Schwab–England	77.8 ± 24		
MDS-UPDRS part III (on)	25.6 ± 15		
Total MDS-UPDRS (I-III) (on)	37.9 ± 26		
MDS-UPDRS part IV (all patients)	1.2 ± 2.2		

Mean ± SD. Statistical comparisons were carried out with the χ^2^ test (dichotomous variables) or the Student’s *t*-test (quantitative variables). Abbrev.: AOPP, advanced oxidation protein products; CSF, cerebrospinal fluid; MDS-UPDRS, International Parkinson and Movement Disorder Society-Sponsored revision of the Unified Parkinson’s Disease Rating Scale; MPO, myeloperoxidase; nd, non-detectable; NS, not significant; *p*, two-tailed probability value; PD, Parkinson’s disease.

**Table 2:** Correlation of MPO concentration and activity in the CSF with demographic and clinical parameters in the cohort of patients with idiopathic Parkinson’s disease.

	MPO Content		MPO Activity	
Parameter	Correlation, r	*p*	Correlation, r	*p*
Age, years	0.298	NS	−0.022	NS
Body mass index	0.102	NS	−0.021	NS
Education, years	0.091	NS	0.076	NS
Albumin index	0.024	NS	−0.129	NS
Leukocyte count, cells/µL	0.123	NS	0.202	NS
Serum MPO concentration (pg/mL)	−0.091	NS	−0.079	NS
Serum MPO activity (mU/mL)	−0.121	NS	0.385	NS
CSF MPO activity	0.471	0.0049		
Levodopa equivalent daily dose, mg	0.037	NS	0.244	NS
Disease duration, years	0.475	0.0045	0.070	NS
Age-at-PD onset, years	−0.156	NS	0.034	NS
Hoehn–Yahr stage	−0.121	NS	0.128	NS
Modified Schwab–England	−0.175	NS	0.028	NS
MDS-UPDRS part III (on)	−0.059	NS	0.045	NS
Total MDS-UPDRS (I-III) (on)	0.010	NS	−0.096	NS
MDS-UPDRS part IV (all patients)	0.044	NS	0.093	NS

Statistical correlation was carried out with the Pearson’s test. Correlation value was adjusted for age, age-a-PD onset, body mass index, and education. Abbrev.: CSF, cerebrospinal fluid; MPO, myeloperoxidase; NS, no significant; MDS-UPDRS, International Parkinson and Movement Disorder Society-Sponsored revision of the Unified Parkinson’s Disease Rating Scale; PD, Parkinson’s disease; *p*, two-tailed probability value.

**Table 3:** Demographic and clinical parameters in the cerebrospinal fluid and serum in men and women.

Parameters	PD		Control	
	Men (*n* = 18)	Women (*n* = 16)	Men (*n* = 13)	Women (*n* = 17)
Age, years	63.2 ± 10	62.1 ± 12	56.9 ± 15	584 ± 12
Body mass index, kg/m^2^	23.1 ± 7	23.3 ± 3	24.4 ± 5	24.9 ± 3
Education, years	17.3 ± 1.3	17.4 ± 1.7	17.5 ± 4	17.3 ± 3
Albumin index	6.8 ± 1.6	7.0 ± 2	6.7 ± 1.5	6.5 ± 1.8
Leukocyte count, cells/µL	3.1 ± 0.6	3.2 ± 0.8	2.8 ± 0.5	2.6 ± 0.7
CSF MPO content, pg/mL	148 ± 63 *	81 ± 71	99 ± 41	103 ± 47
CSF MPO activity, mU/mL	0.0294 ± 0.001	0.0287 ± 0.001	0.0286 ± 0.001	0.0294 ± 0.001
Serum MPO content, pg/mL	3890 ± 3100 *	3690 ± 1695 **	1242 ± 727	1650 ± 608
Serum MPO activity, mU/mL	44.3 ± 3 **	43.7 ± 4 **	35.1 ± 6	35 ± 6
CSF AOPP content, µM	16.7 ± 23 (*n* = 6)	9.9 ± 19 (*n* = 10)	nd	nd
Serum AOPP content, µM	588 ± 311	455 ± 391	nd	nd
Levodopa equivalent daily dose, mg	714 ± 664	650 ± 432		
Disease duration, years	11 ± 8	12 ± 8		
Age at PD onset, years	51.4 ± 13	53.7 ± 15		
Modified Hoehn–Yahr stage	2.2 ± 0.7	2.1 ± 0.8		
Modified Schwab–England	76.5 ± 23	68.8 ± 27		
MDS-UPDRS part III (on)	29.4 ± 14	23.8 ± 17		
Total MDS-UPDRS (I-III) (on)	44 ± 26	37.2 ± 30		
MDS-UPDRS part IV (all patients)	1.4 ± 2.5	1.5 ± 2.2		

Mean ± SD. Statistical comparisons were carried out with the χ^2^ test (dichotomous variables) or the Student’s *t*-test (quantitative variables). * *p* < 0.01 versus PD women, ** *p* < 0.001 versus the corresponding control group. Abbrev.: AOPP, advanced oxidation protein products; CSF, cerebrospinal fluid; MDS-UPDRS, International Parkinson and Movement Disorder Society-Sponsored revision of the Unified Parkinson’s Disease Rating Scale; MPO, myeloperoxidase; nd, nondetectable; NS, no significant; PD, Parkinson’s disease.

**Table 4:** Correlation of MPO concentration and activity in the CSF with demographic and clinical parameters in male and female patients with idiopathic Parkinson’s disease.

	MPO Content		MPO Activity	
Parameter	Men (*n* = 18)	Women (*n* = 16)	Men (*n* = 18)	Women (*n* = 16)
Age, years	0.158	0.405	−0.022	−0.023
Body mass index	0.101	0.087	−0.024	−0.025
Education, years	0.275	−0.364	0.159	−0.306
Albumin index	0.021	0.028	−0.149	−0.103
Leukocyte count, cells/µL	0.101	0.204	0.212	0.186
CSF MPO activity	0.470 *	0.498 *		
Serum MPO content	−0.387	0.216	−0.174	−0.109
Serum MPO activity	0.128	0.326	0.314	−0.273
Levodopa equivalent daily dose, mg	0.016	0.076	0.230	0.331
Disease duration, years	0.469 *	0.565 *	0.404	−0.276
Age-at-PD onset, years	−0.135	−0.024	−0.256	0.329
Hoehn–Yahr stage	−0.048	−0.236	0.343	0.104
Modified Schwab–England	−0.018	−0.401	0.010	−0.006
MDS-UPDRS part III (on)	−0.019	−0.307	0.324	0.013
Total MDS-UPDRS (I-III) (on)	−0.017	−0.183	0.292	−0.120
MDS-UPDRS part IV (all patients)	0.216	−0.012	0.320	−0.080

Statistical correlation was carried out with the Pearson’s test. Correlation value was adjusted for age, age-at-PD onset, body mass index, and education. * *p*< 0.05. Abbrev.: AOPP, advanced oxidation protein products; CSF, cerebrospinal fluid; MPO, myeloperoxidase; NS, no significant; MDS-UPDRS, International Parkinson and Movement Disorder Society-Sponsored revision of the Unified Parkinson’s Disease Rating Scale; PD, Parkinson’s disease.

## Data Availability

Data contained in the article are available upon request.
